# Effects of testing speed on the tensile and mode I fracture behavior of specimens printed through the Fused Deposition Modeling technique

**DOI:** 10.1038/s41598-024-54780-9

**Published:** 2024-02-17

**Authors:** Jiangtao Zhan, Jie Cai, Reza Hasani

**Affiliations:** 1 School of Creative Arts and Design, Zhejiang Institute of Mechanical and Electrical Engineering, Hangzhou, 310053 Zhejiang China; 2 Cryogenic Fluid Equipment R&D Zhejiang Engineering Research Center, Zhejiang Institute of Mechanical and Electrical Engineering, Hangzhou, 310053 Zhejiang China; 3 Islamic Azad University Central Tehran Branch, Tehran, Iran; 4 College of Technical Engineering, The Islamic University, Najaf, Iraq

**Keywords:** Fused deposition modeling, Test speed, Mechanical properties, Fracture resistance, J-integral criterion, Energy science and technology, Engineering

## Abstract

Additive Manufacturing (AM) processes are known as revolutionary manufacturing processes that fabricate a part using a 3D model layer upon layer. These techniques gained more attention from various industries due to their advantages like low waste material. Also, these processes can produce any part with high degrees of complexity in a short period of time. The Fused Deposition Modeling (FDM) process is a material extrusion-based technique which works by extruding a fine molten polymeric filament through a heated nozzle on the heated platform named printer bed. In this method, some important manufacturing parameters play a crucial role in controlling the mechanical properties and quality of the final fabricated part. However, all printed specimens through the FDM process should be tested based on the standards under some critical circumstances. Thus, in the current research paper, five and three test speeds are considered in tensile and fracture testing procedures, respectively to evaluate how these speeds can affect the mechanical and mode I fracture properties. Also, as the FDM specimens present elastic–plastic behavior, the critical value of J-integral is assumed as a fracture assessment and calculated from the finite element analysis. Among the mechanical properties, ultimate tensile strength is affected significantly by the test speed. For instance, the ultimate tensile strength of FDM specimens is 39.02, 38.58, 42.33, 48.09, and 52.11 for test speeds of 2, 4, 6, 8, and 10 mm/min, respectively. But vice-versa results are detected for the mode I fracture behavior and corresponding values of *J* for the FDM-PLA specimens. Finally, experimental and numerical results together with comprehensive discussions about the considered speeds and obtained results are reported.

## Introduction

Additive Manufacturing (AM) technologies are known as 3D printing work by producing any part even with high degrees of geometrical complexity layer upon layer^1^. AM processes are now against the traditional manufacturing techniques including milling, machining, casting, etc. because of some advantages such as low waste material and cost effects^2^. It should be noted that one of the aspects of AM is to design the final part through the use of Computer Aid Design (CAD) and also, some new methods were developed based on the CAD^3^. AM consists of many technologies that are classified into different categories (e.g., powder bed fusion, material extrusion, vat photopolymerization, sheet lamination, direct energy deposition, etc.) and each of them has its own applications and concepts^4^. Fused Deposition Modeling (FDM) is a subcategory of the material extrusion group and in this technique, a fine molten filament is extruded from a heated nozzle with a special diameter on a heated platform (namely printer bed)^5^. Various polymeric filaments are used in the FDM technique the selection of them depends on the application of the final printed part. Acrylonitrile Butadiene Styrene (ABS), Polylactic Acid (PLA), Wood, Polyethylene Terephthalate Glycol (PET-G), and even composite materials are the main used materials in the FDM process^6^. Among the mentioned materials, PLA is the most widely used 3D filament because it is easy to print. This plastic is made from vegetable starches, having the property of being biodegradable and being able to melt at low temperatures^7^. Thus, many industries (e.g., food and dental industries) use this polymer for printing some complex instruments integrally^8^. It should be stated that the PLA material shows fewer changes compared to the ABS material when exposed to high temperatures^9^. However, the FDM-PLA specimens have a significant dependency to the layer thickness in comparison to the FDM-ABS specimens^10^. Besides the input material, some important manufacturing parameters are involved in the FDM process and the effects of them on the final quality of the printed part is obvious. In-plane raster angle^11^, out-of-plane layer orientation^12^, nozzle diameter^13^, nozzle and bed temperatures^14^, infill density, fill pattern^15^, printing speed^16^, wall thickness, and layer thickness^17^ are the main parameters that have a great significance on the mechanical properties of the fabricated part.

In recent years, many researchers have studied the effects of the mentioned manufacturing parameters on the mechanical performance of 3D-printed samples. The building orientation, or the position in which a sample is placed on the platform of the 3D printer, can have a significant effect on the strength of the FDM printed parts^18^. Since this orientation affects the mechanical properties, anisotropic behavior describes FDM printed parts, which is comparable to composites^19^. Better mechanical properties are provided by flat and on-edge building orientations; however, on-edge represents a significant manufacturing challenge due to the limited contact with the printing platform, leading to significant instability and the requirement, depending on the manufacturing complexity^20^. The PLA material's layer thickness, infill orientation, and number of shell perimeters were examined by Lanzotti et al.^21^. In this research, the CCD technique (three parameters) was utilized for analysis. The ultimate tensile strength behavior was variable with layer thickness. According to Ahn et al.^22^, the raster width and bed temperature had only a minor impact on the tensile strength of FDM-ABS parts, whereas the raster orientation had a significant impact. In the study by Yu et al.^23^, increasing the printing speed typically caused a decrease in the tensile strength and compressive strength of PLA parts produced using the FDM process. The plastic materials’ mechanical properties have a strong relation with the testing speed. For this purpose, Ergene and Bolat^24^ concluded that the lower testing speeds resulted in higher plastic behavior and in contrast, higher testing speed resulted in the brittle failure mechanism. It is worth noting that one of the important manufacturing parameters is the infill density and it was proved that the higher infill density resulted in higher impact properties. Thus, this parameter should be considered in different aspects of manufacturing processes^25^.

It is worth noting that many parts may fail under different loading conditions in the presence of a crack or other discontinuity in the industries. Thus, the fracture behavior of the FDM samples under different loading conditions gained more attention from researchers. By employing Single Edge Notch Bending (SENB) specimens, Hart et al.^26^ investigated the impact of layer orientation on the fracture toughness of FDM-ABS parts. In their study, samples that were oriented horizontally had higher fracture loads than samples that were oriented vertically. Additionally, for specimens that were oriented horizontally and vertically, respectively, brittle and ductile fracture behaviors were seen. Aliheidari et al.^27^ used a double cantilever beam to assess the interlayer adhesion and fracture resistance of FDM-ABS specimens. According to the findings, in the FDM-ABS specimens, interlayer fracture resistance was greater than the apparent one.

As stated in this section, different manufacturing parameters were considered as variables and the influence of them was examined. In plastics, testing speed is an important factor due to the viscoelastic behavior of them. Hence, in the current research paper, some FDM specimens were printed and tested at different speeds. Then, tensile properties were extracted and compared to understand how the tensile test speed affected the mechanical properties. Also, mode I fracture experiments were conducted on the FDM specimens with different test speeds. Finally, comprehensive conclusions about the results were reported in this paper.

## Methodology

### Material and printing procedure

As stated in the previous section, commercial polymers like PLA and ABS are mostly used filaments for the FDM process. Thus, here, white PLA filament which was provided from the Filatech^28^ with an initial diameter of 1.75 mm was implemented as an input material. The mechanical and physical properties of this material are listed in Table 1. The manufacturing parameters that were kept constant during the printing process are reported in Table 2. The selection of these parameters was related to the PLA filament catalog. It is worth noting that all the printed specimens through the FDM machine were cooled down for a few minutes on the printer bed after finishing the fabricating procedure. This issue helped to avoid shrinkage of the printed specimens.

**Table 1:** Mechanical and physical properties of PLA material^29^.

Density	1.00–3.41 g/cc
Thermal conductivity	0.0320–0.170 W/m–K
Melting point	64–220 °C
Glass transition temperature (T_g_)	43–120 °C
Modulus of elasticity	0.05–13.8 GPa
Yield strength	8–103 MPa
Ultimate tensile strength	10–3000 MPa

**Table 2:** Manufacturing parameters for printing the PLA specimens.

Nozzle diameter	0.4 mm
Nozzle temperature	215 °C
Bed temperature	55 °C
Infill density	100%
Raster angle	45/–45 °C
Layer orientation	Flat (laid on the bed)
Layer thickness	0.2 mm
Printing speed	55 mm/s

### Geometries

**Figure 1:**
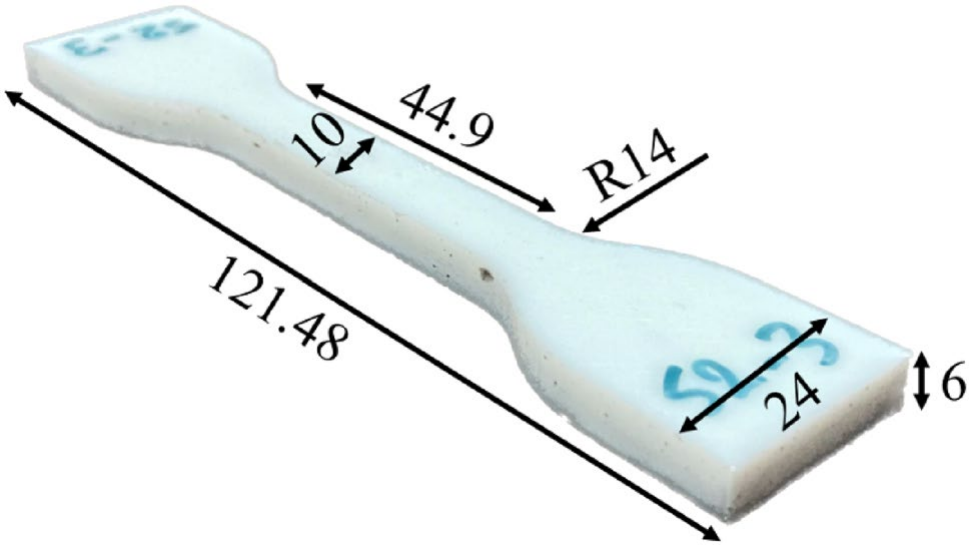
Dog-bone-shaped specimen together with dimensions (all in mm) printed for tensile experiments.

In the current study, two different types of specimens were considered for tensile and fracture experiments. To evaluate basic mechanical properties, a dog-boned-shaped specimen was designed based on the ASTM-D638 (type IV)^30^ and then printed by the FDM technique considering the manufacturing parameters reported in Table 2. A printed dog bone together with the dimensions is depicted in Fig. 1.

**Figure 2:**
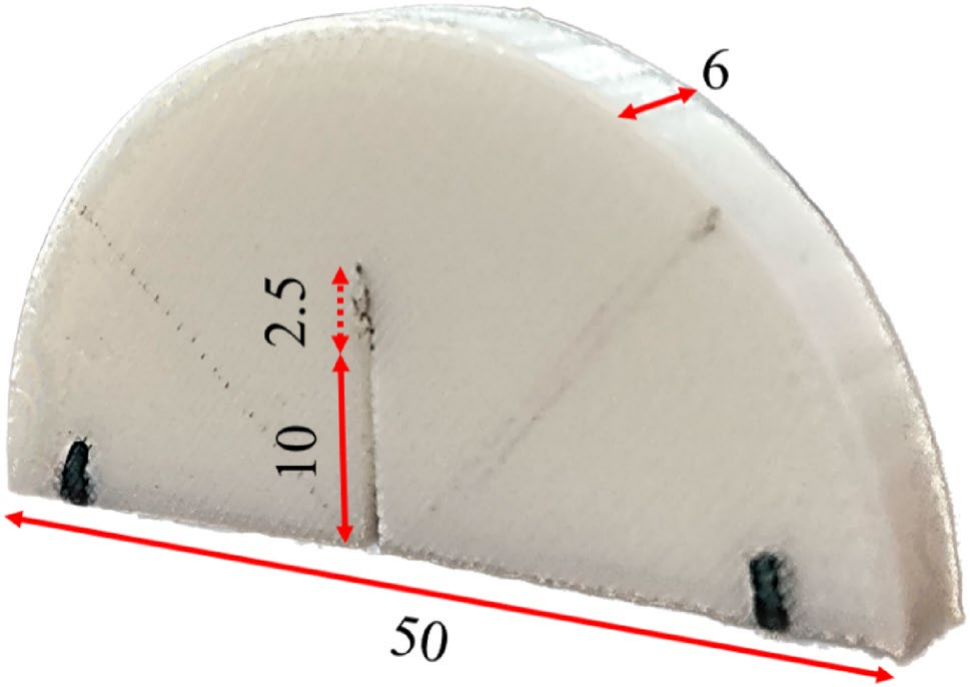
SCB specimen together with dimensions (all in mm) printed with the FDM.

To get deep insight into the fracture behavior of cracked FDM-PLA specimens, some Semi-Circular Bending (SCB) specimens were designed and printed by the FDM technique. This practical geometry is mostly implemented to assess the fracture behavior of brittle materials^31^. SCB specimens had 50 mm diameter (see Fig. 2) and a pre-crack of 12.5 mm was generated in them. To this end, 10 mm of the pre-crack was created by a sawing machine, and 2.5 mm left was produced by a special razor blade as seen in Fig. 2.

### Experiments

**Figure 3:**
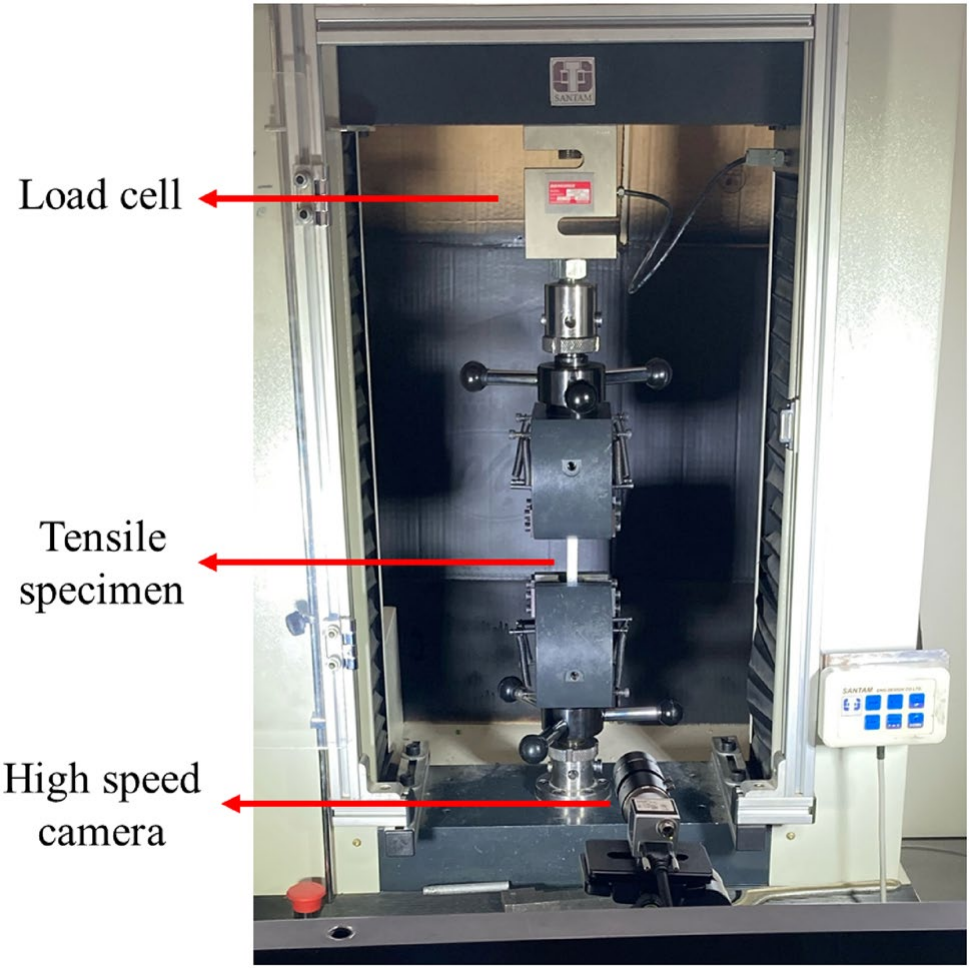
Tensile testing setup together with the DIC equipment.

A universal testing machine (with load capacity of 50 KN) was implemented to conduct tensile tests to obtain basic mechanical properties. Load–displacement data were obtained and then stress–strain curves were plotted. Stress values were calculated by dividing the loads by the cross-section area and strains were evaluated through the use of the Digital Image Correlation (DIC) technique. For this aim, first, speckle patterns were generated on the surface of dog bones, and during the tensile experiments, photos were captured from the tensile specimens with 5s time intervals. Then, by using GOM-Correlate software^32^ strains were obtained. For more information about this technique, see reference^33^. The testing setup together with the DIC equipment are presented in Fig. 3. As stated earlier, the main purpose of the current paper was to investigate the testing speed on the tensile and mode I fracture behavior of FDM-PLA specimens. Tensile experiments were conducted with five speeds (i.e., strain rates) of 2, 4, 6, 8, and 10 mm/min and each tensile test was repeated three times to ensure the repeatability of the experiments, thus, 15 dog bones were printed by the FDM and tested.

**Figure 4:**
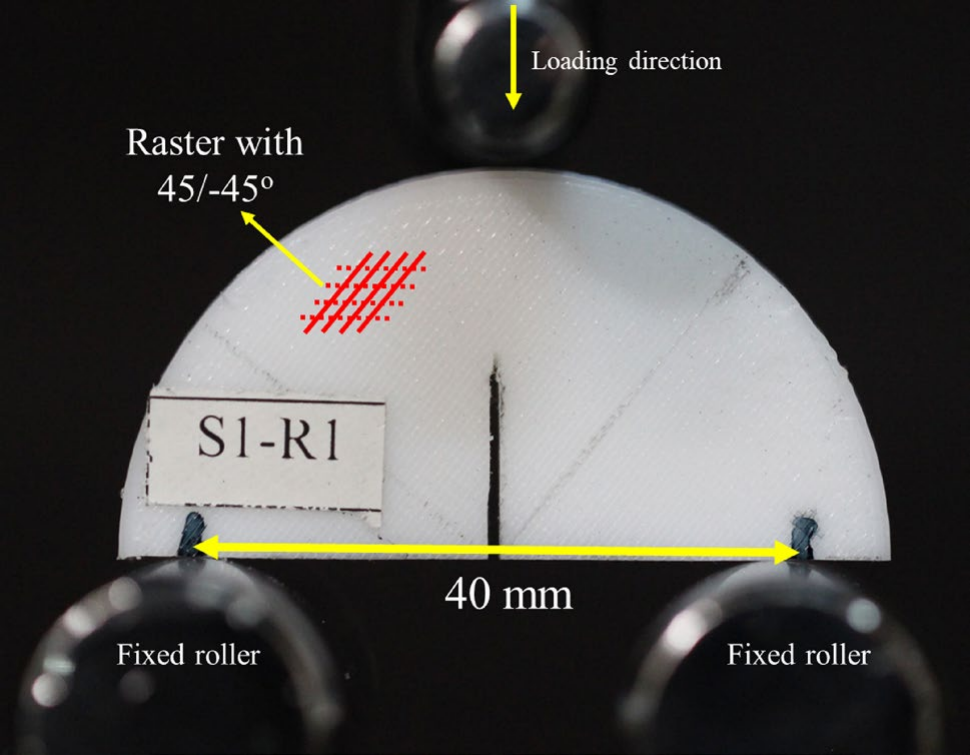
Three-point bending test setup with SCB together with raster orientation.

However, another purpose of this paper was to study the fracture behavior of the FDM specimens under mode I load conditions. SCB specimens were prepared according to the procedures mentioned in section "Geometries" and Fig. 2 and finally, tested based on the three-point bending setup. Three test speeds were considered as 1, 3, and 5 mm/min for the fracture experiments. The reason for selecting the mentioned speeds for the tensile and fracture experiments was to conduct the quasi-static loading condition. On the other hand, in the higher test speeds especially for the tensile tests, some moments might be missed. Each fracture test was repeated three times to ensure the repeatability of the obtained results. A three-point test setup consisted of three rollers where one of them was located on the top of the SCB and others were fixed in the bottom of the SCB specimen. Figure 4 shows the fracture experiment with a three-point bending setup.

It is worth noting that all the experiments were conducted at room temperature.

## Theoretical background

Based on the fracture mechanics, fracture happens when a fracture property reaches its critical value^34^. With this in mind, brittle fracture occurs when the stress intensity factor (that is magnitude of stress around the crack tip vicinity) reaches the fracture toughness (*K*_*Ic*_). But for the materials with elastic–plastic behavior, another parameter named *J* is recommended. This parameter is a rate of change in the net potential energy around the crack tip and is an approximate selection for materials with slight anisotropy. The *J* values can be calculated directly using contour integrals or the virtual crack extension method^35^.

**Figure 5:**
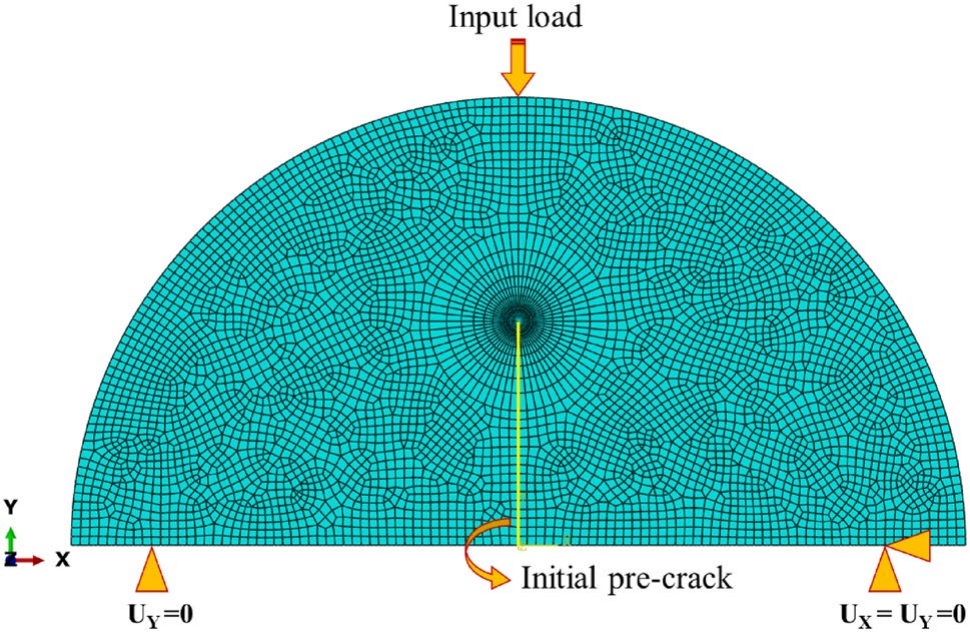
Mesh pattern and loading condition of SCB specimen.

Here, critical values of *J* (i.e., *J*_*c*_) were considered as fracture assessment and they were calculated through the use of the finite element method. For this aim, the SCBs were modeled in finite element software, and mechanical properties that were obtained in the previous section were assumed as mechanical properties for the SCB specimens. It should be noted that the FDM parts are known as anisotropic materials but slight ones, therefore, the isotropic assumption was an appropriate consideration and helped to avoid time-consuming analyses of anisotropic assumption. However, some researchers previously concluded that the FDM specimens could be modeled as homogenous material. One of the studies that recommended isotropic assumption was a research conducted by Domingo-Espin et al.^36^. The obtained results revealed that the anisotropic assumption only could improve the final results with a minor difference compared to the isotropic assumption. The mechanical properties obtained from different five test speeds were averaged and then imported to the finite element simulation procedure. After creating some section on the SCB sample, a 2D plane-stress model with quadratic quadrilateral (element code: CPS8R) elements is considered for the SCB specimens. Then, the SCBs were loaded using the fracture loads obtained from the fracture experiments, and corresponding to the elastic–plastic analysis, values of *J*_*c*_ were evaluated and compared. A mesh pattern and loading condition in the SCB model are illustrated in Fig. 5. It should be noted that mesh convergence analyses also were performed to find a proper number of elements and the SCB specimen contained 5244 elements.

## Results and discussion

### Tensile test results

**Figure 6:**
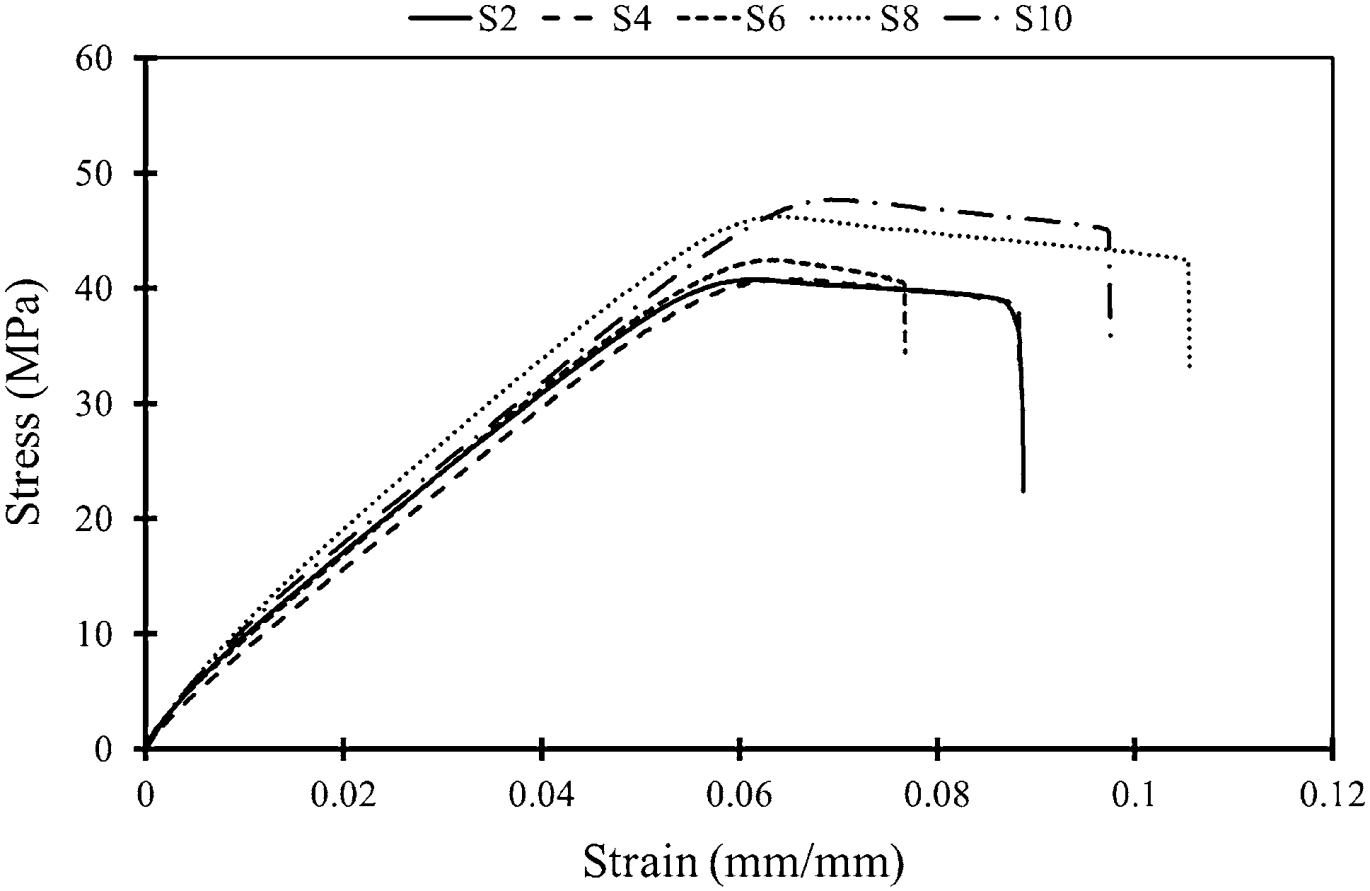
Representative true stress–strain curves of FDM-PLA tensile specimens that were tested with different speeds.

The main purpose of the current research study was to check how the test speed during the experiments could affect the mechanical integrity and mode I fracture behavior. In the first step, the mechanical properties of FDM-PLA specimens were considered and mechanical properties were obtained under different test speed conditions. True stress–strain curves are exhibited in Fig. 6. In this figure, representative curves are shown and it should be noted that in these curves, the DIC technique was implemented to calculate strains in two vertical and horizontal directions. In Fig. 6, S2, S4, S6, S8, and S10 denote test speeds of 2, 4, 6, 8, and 10 mm/min, respectively.

A summary of the mechanical properties is reported in Table 3. In this table, *E*, *υ*, *σ*_*u*_, *σ*_*y*_, and *ε*_*failure*_ are Young’s modulus, ultimate tensile strength, yield strength, and strain at failure, respectively.

**Table 3:** Average mechanical properties of FDM-PLA specimens that tested with different speeds.

Test speed (mm/min)	*E* (MPa) ± STD	*υ* ± STD	*σ*_*u*_ (MPa) ± STD	*σ*_*y*_ (MPa) ± STD	*ε*_*failure*_ ± STD
2	1342 ± 241	0.27 ± 0.004	39.02 ± 2.2	35.66 ± 3.5	0.070 ± 0.002
4	1380 ± 79	0.26 ± 0.005	38.54 ± 5.1	36 ± 2.9	0.072 ± 0.003
6	1506 ± 109	0.28 ± 0.005	42.33 ± 4.5	37.98 ± 3.5	0.063 ± 0.002
8	1873 ± 231	0.28 ± 0.006	48.09 ± 3.4	43.27 ± 3.7	0.115 ± 0.005
10	1751 ± 283	0.29 ± 0.004	52.11 ± 3.5	43.50 ± 4.6	0.102 ± 0.004

The behavior of many different types of materials, including plastics, is governed by the principle that ductility decreases as strength and stiffness increase. The property of ultimate elongation, or elongation at break, in a tensile test, is regarded as a comparative indicator of ductility^37^. According to Fig. 6 and Table 3, the FDM-PLA specimens showed elastic–plastic behavior and plastic deformation was completely obvious in the curves. Also, two test speeds of 2 and 4 mm/min had similar results from a mechanical properties point of view. But as the test speed increased Young’s modulus increased too and the highest value of *E* was related to the test speed of 8 mm/min. Overall, it can be stated that the test speeds of 8 and 10 mm/min presented the highest mechanical properties, thus, selecting the test speed is mostly dependent on the operator and the final aims of the tensile experiment.

As stated in section "Theoretical background", when a material shows elastic–plastic behavior in the experiments, utilizing Linear Elastic Fracture Mechanics (LEFM) theories for examining their fracture behavior is not valid. Therefore, selecting *J*_*c*_ as a fracture criterion in this research paper was a logical selection because this parameter works for both linear elastic and non-linear ones based on fracture mechanics. The presence of plasticity in the FDM-PLA specimens belonged to the two phenomena, one was the raster angle of 45/-45°. Printing specimens with this raster orientation resulted in higher plastic deformation because it helped to increase shear stresses and consequently plasticity. On the other hand, polymers (like PLA which was used as an input material in this paper) have viscoelastic behavior. Thus, test speed affected the time of experiment and loading condition directly and this parameter influenced the mechanical properties.

The equivalence of time and temperature in determining a material's behavior is a key concept in viscoelastic behavior^38^. This rule applies to the mechanical properties of any plastic because all polymers are naturally viscoelastic. Unfortunately, only textbooks that give a very thorough mathematical analysis of the topic tend to address these principles. Engineers who design plastic parts and specify raw materials frequently lose sight of this crucial principle because the math often obscures the practical significance. Essentially, when temperatures are raised under load, the effects of long stretches of time without raising temperatures are also noticeable. The strain rate is the reciprocal of time. While shorter times are associated with higher strain rates, longer times are associated with lower strain rates. Therefore, lower strain rates mimic higher temperature behavior, whereas higher strain rates reflect lower temperature behavior^39^.

### Fracture experiment results

**Figure 7:**
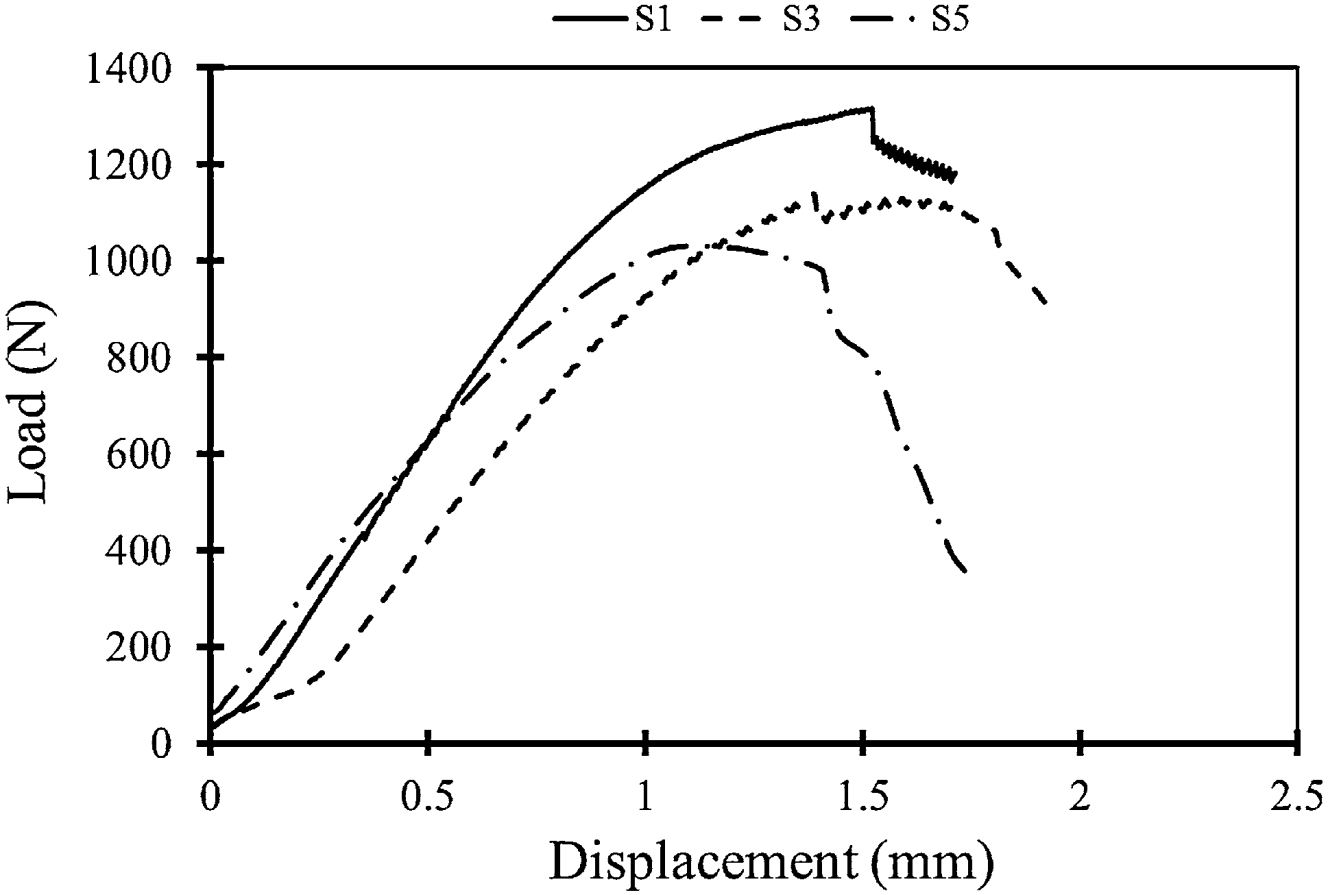
Representative load–displacement curves of FDM-PLA specimens tested with different test speeds.

Mode I fracture experiments were conducted on the SCB specimens that were printed with constant manufacturing parameters. However, all the SCBs were tested with three test speeds of 1, 3, and 5 mm/min. Representative load–displacement curves of FDM-PLA specimens that tested with the mentioned speeds are illustrated in Fig. 7. In this figure, S1, S3, and S5 are related to the test speeds of 1, 3, and 5 mm/min, respectively. A summary of the fracture results is also reported in Table 4.

**Table 4:** Summary of the fracture experiments for the FDM-PLA SCB specimens that tested with three speeds.

Test speed (mm/min)	Fracture loads (N)			Average	Standard deviation
	Repetition 1	Repetition 2	Repetition 3		
1	1380	1315	1177	1290	± 84
3	1372	1138	1100	1203	± 120
5	1031	1034	1181	1082	± 70

Based on Fig. 7 and Table 4, as the test speed increased the fracture load decreased and there were some reasons for this issue. Pursuant to the literature, in the FDM products, there are some weak zones namely inter-raster and inter-layer zones. In most cases, the failure happens in these areas because they do have not much resistance against the fracture extension. When the fracture experiment was conducted fast (i.e., higher test speed), the initial crack had less time to be inserted into the weak spots, and the crack kinking phenomenon was in trouble.

However, when the initial pre-crack tended to grow, the raster angle of 45/–45° in the FDM specimens resulted in crack kinking, thus, the pre-crack faced only half of the deposited filaments (i.e., rasters) perpendicular to them and another half were parallel to them. Thus, inter-raster failure was possible especially when the test speed was 1 mm/min.

**Figure 8:**
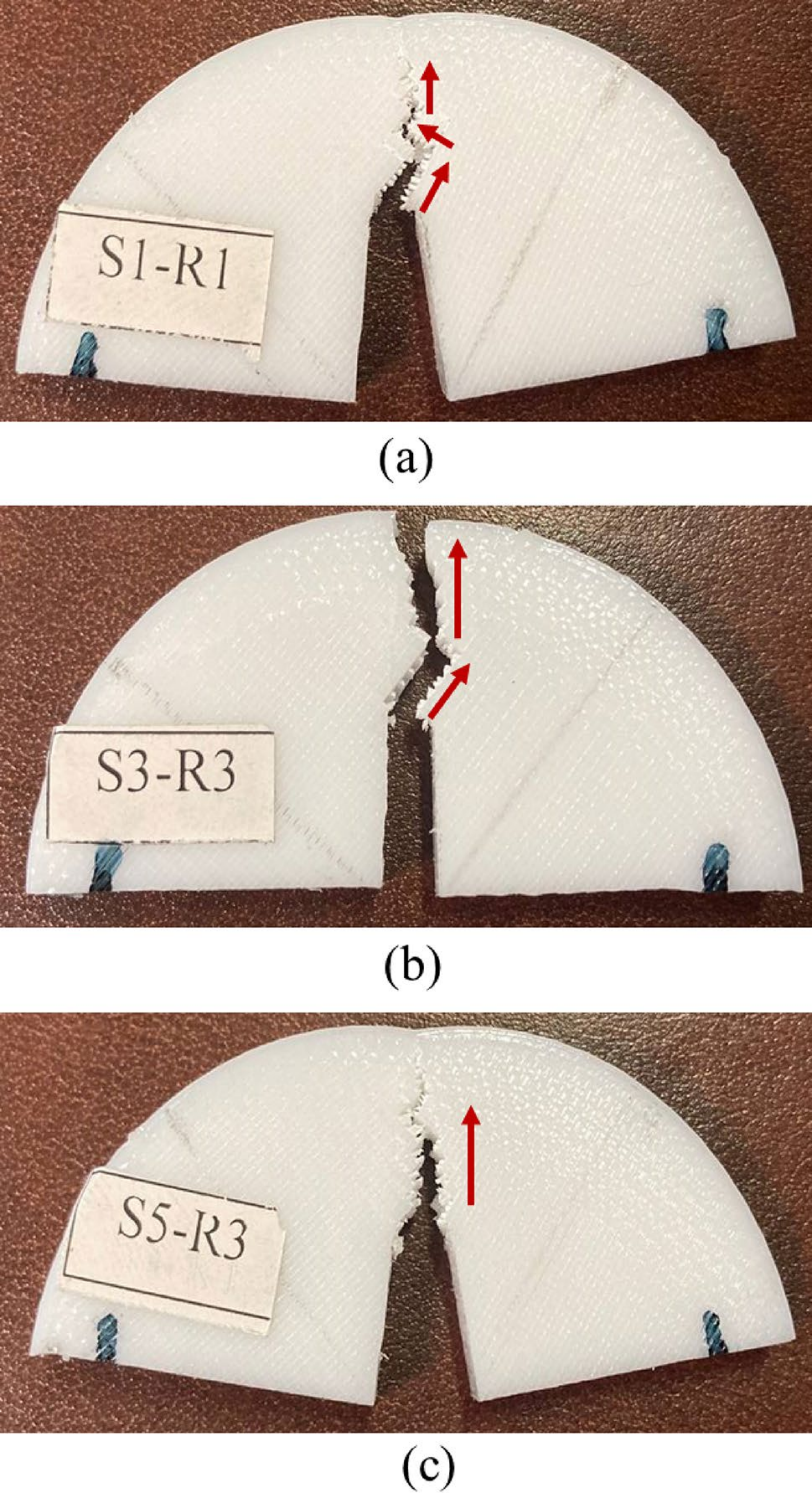
Fractured SCB specimens that printed with a 45/–45° raster angle and tested with speeds of (**a**) 1 mm/min, (**b**) 3 mm/min, and (**c**) 5 mm/min.

As a matter of fact, plastic deformation around the crack and shear stresses are two important factors that play a significant role in increasing the fracture load. When the test speed increased to 3 or 5 mm/min, the initial pre-crack grew fast and plastic deformation around the crack tip vicinity decreased. Overall, it can be stated that although 45/-45° raster orientation in the cracked SCB specimens helped to create shear stresses, but higher test speed resulted in lower amounts of plastic deformation around the crack tip. Fractured SCB specimens that were tested with three test speeds of 1, 3, and 5 mm/min are shown in Fig. 8.

As seen in Fig. 8, when the test speed was 1 mm/min the pre-crack grew in 45° due to the raster pattern and some crack kinking was also observed (see the red arrows in Fig. 8). This crack kinking could improve fracture behavior and increased fracture loads, but at the higher test speeds the crack kinking was decreased, thus, the crack pathed a straight line and resulted in earlier failure.

### Fracture resistance (***J***_***c***_ values) results

**Figure 9:**
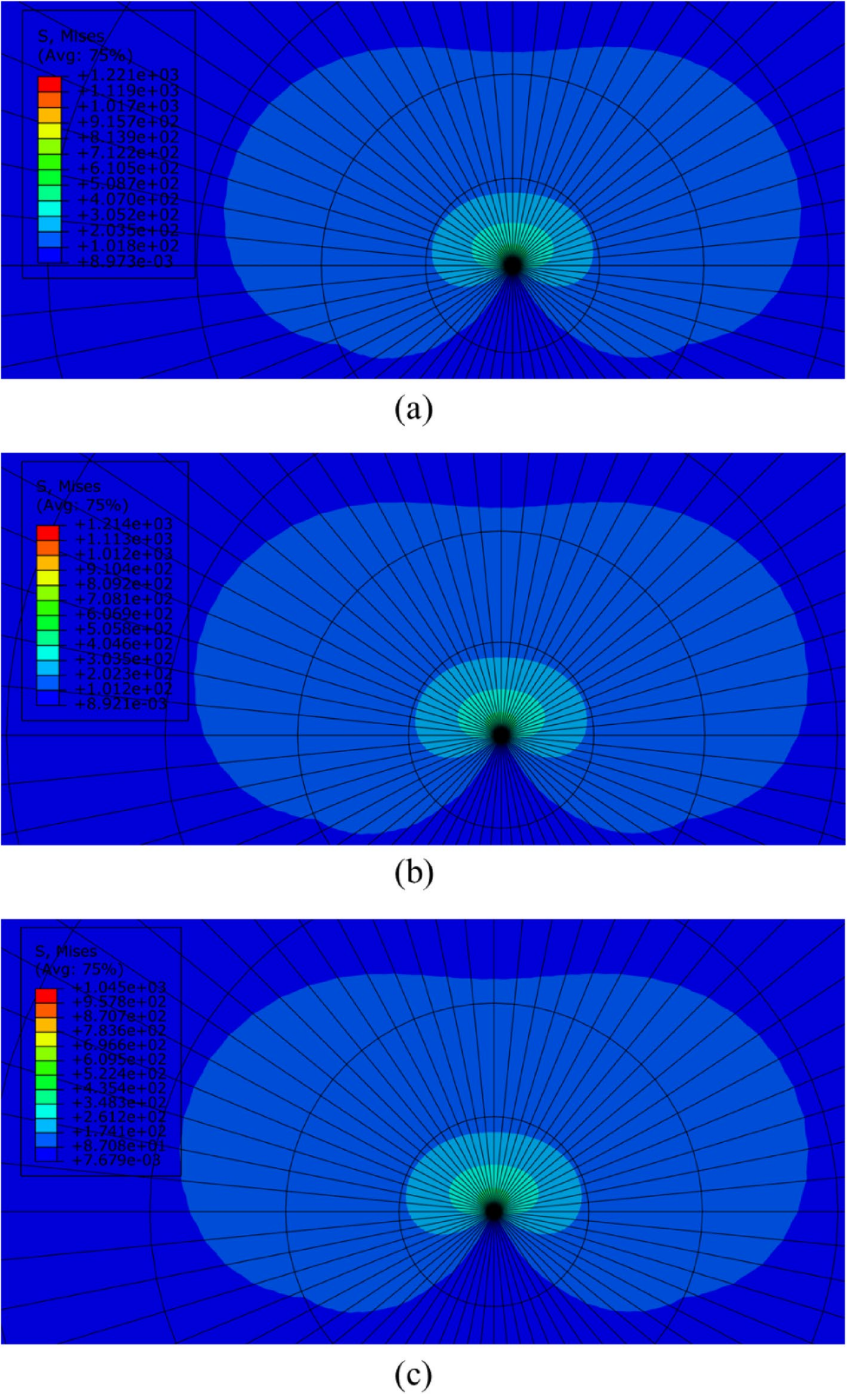
von-Mises stress distribution in the cracked FDM-PLA specimens that tested with three speeds of (**a**) 1 mm/min, (**b**) 3 mm/min, and (**c**) 5 mm/min.

Finite element analysis was implemented to calculate critical values of *J* corresponding to the fracture loads according to the statements presented in section "Theoretical background". To get better insight into the stress distribution in the cracked FDM specimens (SCBs), Fig. 9 presents the von-Mises stress distribution in the SCB specimens that were tested with three different speeds.

According to Fig. 9, the butterfly-shaped stress distribution is obvious which means that all the fracture experiments were done under mode I loading conditions. Also, as the test speed increased the amounts of von-Mises stress decreased too. It should be noted that by considering the plastic deformation size around the crack tip, lower test speed resulted in more plastic deformation. As stated earlier, one of the ways to calculate the critical values of *J* is implementing finite element simulation, thus, after performing finite element analyses, values of *J*_*c*_ were obtained and reported in Table 5.

**Table 5:** Critical values of *J* corresponding to the fracture loads for the cracked FDM-PLA specimens.

Test speed (mm/min)	Fracture load (N)		*J*_*c*_ (J/m^2^)
1	Repetition 1	1380	2371
	Repetition 2	1315	2153
	Repetition 3	1177	1725
3	Repetition 1	1372	2343
	Repetition 2	1138	1612
	Repetition 3	1100	1507
5	Repetition 1	1031	1323
	Repetition 2	1034	1331
	Repetition 3	1737	1737

In Table 5, it is clear that values of *J*_*c*_ decreased as the test speed increased and this was related to the fracture loads. However, this matter was completely obvious but using these values gives a better vision to designers and engineers where they can use FDM-PLA specimens servicing under various conditions. Comparing the obtained results from the finite element analysis here and with the published results in^40^ revealed that the obtained critical values of J-integral were similar to those obtained in^40^ (for the 0.42 mm raster width, it was 2264 J/m^2^).

## Conclusion

Fused Deposition Modeling (FDM) technique is a subcategory of the additive manufacturing processes and nowadays, many industries use this technique for production fabrication. One of the challenges that this technique faces is how to test them according to the standards. Standards suggest various test speeds and selecting them depends on the operator and designer. For this aim, the current research study explored the effects of test speeds on the mechanical properties and mode I fracture behavior of FDM-PLA specimens. During the tensile experiments, the Digital Image Correlation (DIC) technique was used to determine the strains. To evaluate the fracture behavior of these specimens, Semi-Circular Bending (SCB) specimens were printed and tested at various rates. Also, considering the elastic–plastic behavior of FDM-PLA specimens, the critical value of *J* (i.e., *J*_*c*_) was determined to characterize fracture behavior. *J*_*c*_ values were obtained through the use of finite element modeling.

Mechanical properties of FDM-PLA specimens increased as the test speed increased too. There was no difference between the elastic moduli but the ultimate tensile strength was affected directly by the test speed. Higher mechanical properties related to the 8 and 10 mm/min test speed.

Fracture loads were decreased as the test speed increased and to clarify this issue, some phenomena including raster angle, inter-raster zones, and crack growth path were discussed comprehensively. When the test speed increased, the initial pre-crack had not enough time to grow among the raster, and crack kinking did not happen, thus, fracture load decreased. *J*_*c*_ values also were directly related to the fracture loads of SCB specimens, therefore, these values presented a descending trend with an ascending trend of test speed.

Finally, it can be concluded that the results of this paper can be used by designers and engineers to help them in the selection of test speeds for examining the FDM products.

## Data Availability

All data generated or analyzed during this study are included in this manuscript.
